# ‘We're All (Cauliflower) Ears’: A Delphi Study Including Staff and Players to Co‐Construct Sports Science and Medicine (Performance and Wellbeing) Research Priorities for Premiership Rugby

**DOI:** 10.1002/ejsc.70007

**Published:** 2025-07-02

**Authors:** Ben Jones, Omar Heyward, Matt Cross, Keith Stokes, Neil McCarthy, Simon Kemp, Emily Sheppy, Lottie Greenlees, Marc Beggs, Richard Bryan, Jamie Fulton, Rhys Griffiths, Joshua Harris, Rhys Hughes, Sharief Hendricks, Mark Lambert, Karen Jones, Mike Lancaster, Matt Lee, Adam Roberts, Nav Sandhu, Tom Sherriff, Mark Twiggs, Gregory Roe

**Affiliations:** ^1^ Carnegie Applied Rugby Research (CARR) Centre Carnegie School of Sport Leeds Beckett University Leeds UK; ^2^ Premiership Rugby London UK; ^3^ Division of Physiological Sciences Department of Human Biology Faculty of Health Sciences University of Cape Town Cape Town South Africa; ^4^ School of Behavioural and Health Sciences Faculty of Health Sciences Australian Catholic University Brisbane Australia; ^5^ England Performance Unit Rugby Football League Manchester UK; ^6^ Rugby Football Union Twickenham UK; ^7^ Centre for Health and Injury and Illness Prevention in Sport University of Bath Claverton Down UK; ^8^ UK Collaborating Centre on Injury and Illness Prevention in Sport (UKCCIIS) University of Bath Claverton Down UK; ^9^ London School of Hygiene and Tropical Medicine London UK; ^10^ Leicester Tigers Rugby Union Club Leicester UK; ^11^ The Rugby Players Association London UK; ^12^ Exeter Chiefs Rugby Union Club Exeter UK; ^13^ Newcastle Falcons Rugby Union Club Newcastle UK; ^14^ Bath Rugby Union Club Bath UK; ^15^ Gloucester Rugby Union Club Gloucester UK; ^16^ Harlequins Rugby Union Club Twickenham UK; ^17^ Northampton Saints Rugby Union Club Northampton UK; ^18^ Bristol Bears Rugby Union Club Bristol UK; ^19^ Sale Sharks Rugby Union Club Manchester UK; ^20^ Saracens Rugby Union Club London UK

**Keywords:** injury and prevention, methodology, performance, policy, talent

## Abstract

Sports invest in research to optimise performance and enhance athlete wellbeing. Involving stakeholders allows research priorities to be determined, maximising the adoption and relevance of research findings. A three‐round modified Delphi process was used to establish wellbeing and performance research priorities for Premiership Rugby (Professional men's rugby union competition in England). Up to 10 research priorities were provided during Round 1 (grouped into higher‐order categories and themes via content analysis). In Rounds 2 and 3, participants ranked higher‐order categories on a one to five Likert scale. Consensus was defined as ≥ 70% agreement. Sixty‐five participants responded in Round 1 (41 and 32 in Rounds 2 and 3). Staff and player experience of working or playing in the Premiership was 11.0 (4.5–16.5) and 7.0 (6.0–8.5) years. Following Round 1, 393 research priorities were provided and 53 higher‐order research priorities and 26 categories were identified, within three themes: performance, wellbeing and injury. Following Round 3, 21 research priorities reached consensus within performance (*n* = 7), wellbeing (*n* = 6) and injury (*n* = 8). Research priorities for a professional sports league, were established by the application of a pragmatic research lens, to ensure priorities were practically minded and also developed with minimal resource requirements, minimal burden for participants and in a short amount of time, which can be applied in other leagues. Research priorities deemed feasible and lacking a relevant evidence base can be addressed in future studies to maximise impact and compliment the ongoing research programmes already established by the professional league and governing body.

## Summary

- Research in sport should be athlete‐centred and benefit those involved. Involving stakeholders in the identification of research priorities can increase the practical relevance of the research, increasing buy‐in and the adoption of the findings.
- In a professional rugby union league, following a three‐round modified Delphi process, 21 research priorities reached consensus within three themes. Seven were performance, six were wellbeing and eight were injury. The research priorities deemed feasible and lacking a relevant evidence base can be addressed in future studies.
- Research priorities for a professional sports league, were established by the application of a pragmatic research lens, to ensure priorities were practically minded and also developed with minimal resource requirements, minimal burden for participants and in a short amount of time, which can be applied in other leagues.

## 1 Introduction

Governing bodies and professional leagues invest significant resources to undertake research with the aim of optimising performance and enhancing athlete wellbeing. There are numerous past and ongoing funded research projects within professional rugby union in England, which contribute learnings to both the domestic competition and also the global game. For example, the Professional Rugby Injury Surveillance project (PRISP) is the longest running injury surveillance project in global rugby union, with the first study published in 2005 (based on data collected between 2002 and 2004) (Brooks et al. 2005a, 2005b). More recently, the PRISP has resulted in a 16‐year evaluation of concussion rates (West, Cross, et al. 2021) and match injuries (West, Starling, et al. 2021) and an evaluation of training injuries based on 1.5 million hours of exposure (West et al. 2020). This project and subsequent studies (Cross et al. 2019; Stokes et al. 2021) have led to numerous policy changes (Stokes et al. 2021; Williams et al. 2017, 2023) for the benefit of rugby union players in England.

Research in sport should be athlete‐centred and benefit the preparation, performance and wellbeing of athletes (Coutts 2017; Brocherie and Beard 2020). Research in sport should also consider practitioner experience and expertise (Houser and Oman 2010; Jones et al. 2019), with the aim of informing policy and/or driving evidence‐based practice and decision‐making (Coutts 2017; Brocherie and Beard 2020). Although evidence‐based practice should be a key influence on the standards by which practitioners operate, there are multiple barriers to its implementation in sport (Fullagar, McCall, et al. 2019). One major barrier that warrants attention is the perceived lack of relevant and applicable research (Schwarz et al. 2021; Fullagar, Harper, et al. 2019). By actively involving stakeholders and end‐users who are affected by the research processes and outcomes (Jones et al. 2019; Finch et al. 2016; Hendricks 2021), relevant research questions along with feasible methods and study designs can be established (Frisch et al. 2020). Furthermore, multistakeholder‐derived research priorities can be used to prioritise the often‐limited funding available for research in elite sport (Cardinale 2017). To understand the research priorities of multiple stakeholders, well‐established methods, such as the Delphi technique, has proved meaningful (Heyward et al. 2022; Brislane et al. 2022).

Premiership Rugby is the professional men's rugby union competition in England and has strategically committed to investing in player wellbeing alongside the Rugby Football Union (RFU; The national governing body for rugby union in England), Rugby Players Association (RPA; The representative body of elite rugby players in England) and World Rugby (WR; The international governing body for rugby union). To capture the perspectives of a range a stakeholders working or playing in the Premiership, the present study aimed to generate research priorities from the perspectives of staff and players within Premiership Rugby via a modified Delphi method.

## 2 Methods

### 2.1 Research Philosophy

The current study was philosophically underpinned by pragmatism, meaning that it was primarily undertaken with the intention of producing practically meaningful knowledge (Giacobbi et al. 2005). Contrasting traditional research paradigms (e.g., positivism and constructivism), pragmatism does not take a particular ontological or epistemological stance (Morgan 2014). Rather, pragmatism asserts that research should be focused on generating knowledge that provides practical solutions to an a priori identified problem (Morgan 2014). Therefore, pragmatists place their questions at the heart of the research enquiry and select an epistemological anchor and methods that are most suited to providing appropriate solutions (Morgan 2007). In the present study, we aimed to generate insight to develop research priorities derived from the perspectives of staff and players within Premiership Rugby.

Accordingly, for this study, the research team utilised a modified Delphi method following the design and reporting recommendations (Spranger et al. 2022). This was deemed to be the most appropriate method to collect participant perspectives and establish consensus (Heyward et al. 2022; Brislane et al. 2022; Pelletier et al. 2022). Additionally, content analysis was implemented as a process of identifying patterns within the perspectives provided by the participants (Elo and Kyngäs 2008; Graneheim and Lundman 2004).

### 2.2 Study Design

A three‐round modified Delphi process (Heyward et al. 2022; Brislane et al. 2022; Pelletier et al. 2022) was used to establish player wellbeing (Health Promotion Glossary of Terms, 2021; Giles et al. 2020) and performance research priorities from the perspectives of staff (first team and academy) and senior players working or playing within Premiership Rugby. In the first round of the Delphi, this process involved participants providing up to 10 research priorities in the areas of player wellbeing and performance (Table 1), which were subsequently grouped into higher‐order categories and themes via content analysis (Elo and Kyngäs 2008; Graneheim and Lundman 2004). In Rounds 2 and 3 of the Delphi, participants ranked the higher‐order categories from very low to very high priority on a one to five Likert scale. Consensus was defined as ≥ 70% agreement (Heyward et al. 2022; Verhagen et al. 1998; van der Horst et al. 2017). The study received institutional ethics approval, and all participants provided consent.

**TABLE 1:** Definitions and examples used in the questionnaire.

Research definition	The process of studying something to discover new information or reach a new understanding.
Wellbeing definition	A positive state experienced by individuals. Similar to health, it is a resource for daily life and is determined by social, economic and environmental conditions. It encompasses quality of life as well as the ability of people to contribute to the world in accordance with a sense of meaning and purpose (adapted from WHO, (2021)). Components of wellbeing include emotional (positive/negative emotional states, e.g., happiness, stress and anxiety), mental (e.g., purpose, resilience and achievement), social (e.g., relationships and social integration/acceptance) and physical (e.g., general physical health, injuries and financial and living circumstances) (adapted from Giles et al. (2020)).
Wellbeing research priority example	An investigation into the stressors experienced by players in the premiership.
Performance definition	Anything that contributes to how well a player or team can compete in match play.
Performance research priority example	An investigation into the transfer of tackle technique training to match scenarios.

### 2.3 Participants

Current academy or senior team staff and senior players at the 10 Premiership Rugby clubs were invited to participate. To ensure that participants had adequate knowledge of Premiership Rugby and professional rugby union more broadly, staff were required to have a minimum of 3 years of experience working in Premiership Rugby only or a minimum of 1 year experience working at the international level combined with 1 years' experience in the Premiership. Additionally, players were required to have a minimum of 5 years of experience playing in Premiership Rugby, or have been selected in a match‐day squad at the international level at least once, combined with a minimum of 3 years of experience playing in the Premiership. These criteria were defined following consultation with Premiership Rugby, the RPA and the RFU to ensure the perspectives of all broader stakeholders were reflected. Often, a key challenge in Delphi studies is the identification of an ‘expert’ panel (Spranger et al. 2022). However, in the present study, the aim was to establish research priorities by gathering and analysing as many experienced Premiership Rugby support staff and player perspectives as possible; therefore, the participants recruited were deemed to be experts for the purpose of this study.

### 2.4 Delphi Technique

#### 2.4.1 Round 1: Establishing Research Priorities in Premiership Rugby

Prior to initiation, BJ and KS presented an outline of the research project to senior staff from each club. Following this, an email was sent to all heads of department (e.g., performance or medical) at each eligible club detailing the project along with a request to recruit a club research representative (the individual responsible for coordinating the research within their respective club). Once the club research representatives were appointed, each were sent an additional email containing the survey for distribution to players and practitioners within their club who met the inclusion criteria. Additionally, the RPA encouraged their members to complete the survey via their club representatives.

Each participant that met the inclusion criteria received an email‐embedded link to an online questionnaire (Table S1) hosted by Qualtrics (Qualtrics, Washington, USA). In Section 1, the questionnaire included questions regarding contact information, demographic information (job title/playing position, ethnicity, age and education) and questions pertaining to work/playing experience in Premiership Rugby and international rugby union settings. In Section 2, participants were asked to provide up to 10 research priorities in the areas of player wellbeing and performance. Definitions for research, wellbeing and performance and an example research priority for both wellbeing and performance were provided (Table 1). Participants were given 2 weeks to respond to the questionnaire, with reminder emails sent at 7, 10 and 13 days into the 2‐week response period.

### 2.5 Data Analysis

In order to group the research priorities provided by the participants in round One, 205 into higher‐order categories and themes, inductive content analysis was used to group the research priorities into higher‐order categories and themes (Elo and Kyngäs 2008; Graneheim and Lundman 2004). Initially, authors GR and OH immersed themselves in the responses and independently coded the data. Using the process of abstraction, each author then generated subcategories, categories, and finally, themes, that shared common features (Elo and Kyngäs 2008; Graneheim and Lundman 2004). Authors GR and OH then compared their results until both were satisfied that the subcategories (higher‐order research priorities), categories (groupings of higher‐order research priorities) and themes (groupings of categories) accurately represented the raw data.

### 2.6 Rounds 2 and 3: Establishing Consensus on Research Priorities in Premiership Rugby

#### 2.6.1 Round 2

A second online questionnaire (Qualtrics, Washington, USA) containing the previously established higher‐order research priorities, categories and themes was sent to all participants who completed Round 1. Participants were asked to rank each higher‐order research priority on a Likert scale of 1–5 (1: very low priority, 2: low priority, 3: medium priority, 4: high priority and 5: very high priority) (Heyward et al. 2022). An opportunity to add any additional research priorities at the end of the questionnaire was also provided. Participants were given 2 weeks to respond to the questionnaire and were sent reminders at 7, 10 and 13 days into the 2‐week response period.

To assess consensus, Likert scale responses were combined to form 3 agreement categories (i.e., low: 1 and 2, medium: 3 and high: 4 and 5) (Heyward et al. 2022; Zambaldi et al. 2017). Agreement data were considered ordinal, items arranged in a ranked order and thus descriptive statistics for the median and interquartile range were calculated (Joshi et al. 2015). Consensus was defined as ≥ 70% agreement in a specific category (Heyward et al. 2022; Verhagen et al. 1998; van der Horst et al. 2017).

#### 2.6.2 Round 3

In Round 3, participants who completed Round 2 were provided feedback regarding the results. This included a list of the higher‐order research priorities that reached consensus and a further list of those that did not reach consensus with the accompanying median for each respective response. Participants were asked to reflect on their previous rating and re‐rate any higher‐order research priorities (via an online questionnaire) that did not reach consensus in Round 2. Participants were given 2 weeks to respond and were sent reminders at 7, 10 and 13 days into the 2‐week response period.

## 3 Results

### 3.1 Overview of Participants

Sixty‐five participants responded in Round 1, of which 41 and 32 responded in Rounds 2 and 3. Participants represented a broad range of stakeholders, including (Table 2) coaching, medical, psychology, strength and conditioning, sport science, working at the senior and academy levels, in addition to players. The proportion of staff who were in leadership roles (e.g., Director, Head of Department and Manager) was 29% in Round 1, 42% in Round 2% and 53% in Round 3. Staff characteristics were as follows: age: (median [interquartile range]) 36.0 (30.0–41.8) years; sex: 15% female and 85% male; experience working in the Premiership: 11.0 [4.5–16.5] years and education: 63% postgraduate degree, 33% undergraduate degree, 2% A‐levels and 2% BTEC diploma. Player characteristics were as follows: age: 28.0 (26.5–29.0) years, playing experience in the Premiership: 7.0 [6.0–8.5] years and education: 25% postgraduate degree, 25% undergraduate degree and 50% A‐levels.

**TABLE 2:** Participants who responded in Rounds 1, 2 and 3, and their primary role.

	Specific role	Round 1 (research priorities)		Round 2 (consensus)		Round 3 (final consensus)	
		Senior	Academy	Senior	Academy	Senior	Academy
Coaching		4	7	2	6	2	5
Medical	Doctor	4	1	3		3	
	Physiotherapist	13	2	12		12	
	Sports rehabilitator/sports therapist	3		1			
Psychology		1	1		1		
Strength and conditioning		12	3	7	2	5	2
Sport science		2				1	
Player	Back	6		4		1	
	Forward	6		3		1	
TOTAL		**51**	**14**	**32**	**9**	**25**	**7**
		**65**		**41**		**32**	

*Note:* The bold text are the totals.

### 3.2 Round 1: Establishing Future Research Priorities in Premiership Rugby

A total of 393 research priorities (performance *n* = 181 and wellbeing *n* = 212) were provided from which 53 higher‐order research priorities and 26 categories were identified (Table 3). Furthermore, although participants were originally asked to provide research priorities with respect to performance and wellbeing, 3 themes were identified: performance, wellbeing and injury (Table 3).

**TABLE 3:** Research priorities that reached consensus and distribution (%) of votes across the three categories along with the median response and associated interquartile range (IQR).

Category	Higher‐order research priority	Low‐medium‐high (%)	Median response (IQR)
Theme; Wellbeing			
Mental resilience and robustness	The factors that influence mental resilience and robustness and their association with work capacity and performance	3‐13‐84	High (H‐VH)
Medical	The prevalence of health disorders in the premiership (e.g., sleep disorders and mental illness)	8‐11‐82	High (H‐VH)
^a^Medical	The factors that influence and explore the long‐term health of retired professional players (e.g., brain health and musculoskeletal health).	13‐11‐77	High (H‐VH)
Fatigue and recovery	The effect of sleep on injury, illness and stress.	0‐26‐74	High (M‐H)
Psychology	The psychological support needs of players in academy and first team settings (e.g., performance and clinical) and the optimal methods for catering to large numbers of players.	11‐18‐71	High (M‐H)
Transitions	The effects of transitioning between levels (e.g., school to academy, academy to first team, post‐rugby career and moving club) and the potential support mechanisms required.	11‐18‐71	High (M‐H)
Theme; Performance			
^a^Talent identification	The factors associated with progression from academy to playing and performing in the premiership.	2‐11‐87	High (H‐VH)
Organisational behaviour	Factors associated with high performing teams (e.g., team cohesion and leadership) and their relationship with performance.	3‐11‐87	High (H‐VH)
Staff development and provision	Provision and continued development practices of coaching staff.	11‐5‐84	High (H‐H)
Training characteristics	The association between training characteristics (content, volume, intensity and time) and performance.	0‐24‐76	High (H‐H)
Psychology	Performance‐related psychological factors (e.g., pre‐game anxiety, motivation and decision‐making under pressure) and potential psychological interventions.	11‐16‐74	High (H‐H)
Match characteristics	Knowledge and understanding of match characteristics by combining data (e.g., iMG, GPS and performance analysis).	0‐26‐74	High (H‐H)
^a^Fatigue and recovery	The duration to recover from particular types of training (e.g., contact training and forwards units).	6‐21‐72	High (M‐VH)
Theme; Injury			
Risk factors	Targeted interventions for reducing common injuries in the premiership (e.g., calf capacity and soleus injury).	3‐8‐89	High (H‐H)
Risk factors	The association between player physical characteristics (e.g., neck strength) and injury.	3‐13‐84	High (H‐H)
Head impacts	Understanding of iMG data to aid decision‐making (e.g., normative match and drill data).	3‐21‐76	High (H‐VH)
Concussion	The association between neck strength and concussion	8‐18‐74	High (M‐VH)
Risk factors	The association between acute changes in training load metrics (e.g., accelerations/decelerations and total distance) and injury.	3‐24‐74	High (H‐H)
^a^Concussion	Mechanisms of concussion (e.g., tackle) and potential prevention strategies (e.g., match rule changes and contact technique practice) and their effects.	4‐23‐72	High (M‐H)
Return to play	Average return to play times of injuries using standardised criteria (e.g., BAMIC) and the sharing of return‐to‐play case studies.	13‐16‐71	High (M‐H)
Wellbeing	The effects of injury on wellbeing and factors that may have the greatest impact (e.g., severity, chronicity and repeated injury) and develop appropriate support mechanisms.	11‐16‐74	High (H‐H)

Abbreviations: BAMIC = British Athletics Musculoskeletal Injury Classification, H = high, iMG = instrumented mouthguard, IQR = interquartile range, M = medium and VH = very high.

^a^ Indicates a subcategory that reached consensus in Round 1.

### 3.3 Rounds 2 and 3: Establishing Consensus on Future Research Priorities in Premiership Rugby

Four higher‐order research priorities reached consensus (Table 3, denoted by a) whereas 49 remained inconclusive. Following Round 3, an additional 17 higher‐order research priorities reached consensus (Table 3), bringing the total to 21 (40%) overall. Those that did not reach consensus are presented in Table S2.

## 4 Discussion

This study established research priorities for a professional sports league, from the perspectives of staff and players, which is important for both idea generation and adoption and translation of research. The involvement of stakeholders in the research process ensures that research objectives are aligned to the stakeholders' needs and context, thereby increasing the likelihood of successful implementation and impact (Jones et al. 2019; Fullagar, McCall, et al. 2019; Hendricks 2021). Thus, the 21 research priorities identified here within the themes of wellbeing (*n* = 6), injury (*n* = 8) and performance (*n* = 7) can be addressed to maximise impact, complimenting the established ongoing research programmes primarily focused on policy change initiates determined by the governing body and professional league (e.g., injury surveillance or head acceleration events and match limits) (Williams et al. 2023; Sawczuk et al. 2024).

Stakeholders provided up to 10 research priorities in the areas of both player wellbeing and performance, which resulted in three distinct themes; wellbeing, performance and injury. Round 1 research priorities were developed by a 65‐member panel in which coaching, strength and conditioning, medical staff and players were similarly distributed, but psychologists and sport scientists were relatively underrepresented, whereas no nutritionists or analysists responded (Table 2). Both players (11.0 [4.5–16.5] years) and staff (7.0 [6.0–8.5] years) had substantial experience working or playing in the Premiership, whereas a high proportion had an educational background of degree or higher (staff 96% and players 50%), which is a strength of the study.

It was beyond the scope of this study and its methods to establish stakeholders understanding and/or perceptions of the current evidence base. If stakeholders were aware of the current literature, broadly there could be four explanations when aligning the research priorities with the current published evidence base. (1) The topic is a priority and there is a limited/no evidence base, (2) the topic is a priority and the current evidence base does not answer the specific question, (3) the topic is a priority, but due to methodological and/or technological limitations, currently the research cannot answer the question or (4) even in a presence of a strong evidence base, the topic remains a priority given its significance. It is also possible that stakeholders were not fully aware of the rugby union evidence base.

Within the 21 research priorities, which achieved consensus, the highest proportion were within the injury theme (Table 3). Over 70% of the research priorities within the injury theme achieved consensus (Table S2), despite a relatively diverse expert group (Table 2). In contrast, approximately 30% of the research priorities within the performance and wellbeing theme achieved consensus. This is likely due to the relatively high injury risk of rugby union compared to other sports (West, Starling, et al. 2021) as well as the performance consequences (e.g., player availability) (Williams et al. 2016), in addition to the long‐term and ongoing injury surveillance and prevention research (Cross et al. 2016), leading to a cross‐discipline and collective approach to injury prevention (Hendricks et al. 2023). Similarly, four research priorities were specifically related to concussion, head impacts or long‐term brain health (Table 3). Alongside the relatively high rates of concussion in rugby union (West, Cross, et al. 2021), there has recently been an increased focus on concussion and head impacts (Alexander et al. 2023). For example, instrumented mouthguards are now mandated in elite rugby union (Sawczuk et al. 2024), despite relatively poor stakeholder buy‐in (Roe et al. 2024). There are also a number of studies showing the potential association between head acceleration events and/or concussions and negative longer‐term health outcomes (Daneshvar et al. 2023; Stewart et al. 2023), which may also explain why these research priorities were identified.

Of the specific research themes identified, there are a number of ongoing research studies within the Premiership, which may provide a useful start point for either knowledge translation to stakeholders or evidence base to build upon. These include long‐term health of rugby players (Zimmerman et al. 2024), talent identification, specific injury risk factors (Lee et al. 2023), head acceleration events (Sawczuk et al. 2024) and concussion (West, Cross, et al. 2021; Cross et al. 2019; Stokes et al. 2021).

The method implemented in the present study represents an efficient way of determining stakeholder research priorities. A traditional Delphi approach would have required significant resource to systematically search, summarise and appraise the vast body of rugby union literature prior to engaging with stakeholders. Furthermore, it would have placed significant burden on players and practitioners to appraise these summaries themselves, which may have compromised the response rate. Instead, research priorities were ascertained based on players' and practitioners' present understanding of their professional contexts, including any potential knowledge of associated research. These 21 priorities (Table 1) can be assessed by organisations (e.g., professional league and/or governing bodies) and researchers to determine the feasibility (e.g., available funding and other resources and achievable methodologies in rugby union environments). Those that are feasible can be subjected to focused literature reviews to determine if stakeholder research questions have been previously and appropriately answered. Within the context of this study, where robust scientific evidence already exists, that is, applicable to applied practice in rugby union, appropriate knowledge translation and dissemination practices can be utilised (Bartlett and Drust 2021). Dialogue between researchers and stakeholders can ensue to determine if the research is sufficient or if more is still required. Where literature reviews identify that further research is currently needed, researchers and funding bodies can work together to allocate appropriate resource and undertake the research. Furthermore, where research priorities did not research consensus, these specific topics may be important for some but not all stakeholders, therefore may also be appropriately appraised.

### 4.1 Limitations

Although this study provides important information regarding stakeholder perspectives on research priorities, it has some limitations. Although the distribution of first‐team strength and conditioning, coaching, medical staff and players were similar in Round 1, only a small number of sport scientists and psychologists (two from each cohort) participated. Moreover, academy staff were underrepresented, and no nutritionists or analysts from either first‐team or academy responded at all. Also, the study did not establish the number of available participants for each specific discipline (e.g., to establish the response rate for each discipline). As such, the higher‐order research priorities developed in Round 1 may not be representative or balanced for all targeted cohorts. Furthermore, there was a significant dropout rate by Round 3 (overall retention rate: 49%), especially with respect to players (only 2 out of 12 responded). As such, consensus of higher‐order research priorities may not fully reflect the views of all cohorts considered. Finally, the participants in this study did not appear to be aware of the current evidence base, which may have informed research priorities.

## 5 Conclusion

This study aimed to establish research priorities for a professional sports league from the perspective of relevant stakeholders. This was achieved by the application of a pragmatic research lens to ensure priorities were practically minded and developed with minimal resource requirements, minimal burden placed on participants and in a short amount of time (approximately 12 weeks). Across the themes of performance, injury and wellbeing, 21 research priorities reached consensus, whereas 49 remained inconclusive. Research priorities deemed feasible and lacking a relevant evidence base can be addressed in future studies by researchers and funding bodies to maximise impact and compliment the ongoing research programmes already established by the professional league and governing body.

## Conflicts of Interest

All authors, except G.R. and S.H., are employed by professional rugby clubs, professional leagues, players associations or governing bodies, therefore could be perceived to have a conflict of interest.

## Supporting information

Table S1

Table S2

## References

[ejsc70007-bib-0001] Alexander, F. , R. Tucker , B. Jones , and S. Hendricks . 2023. “X as a Proxy for Tackle Safety Culture? Sentiment Analysis of Social Media Posts on Red‐Carded and Yellow‐Carded Tackles During the 2019 Rugby World Cup.” BMJ Open Sport & Exercise Medicine 9, no. 4: e001756. 10.1136/bmjsem-2023-001756. PMC10603336 37901749

[ejsc70007-bib-0002] Bartlett, J. D. , and B. Drust . 2021. “A Framework for Effective Knowledge Translation and Performance Delivery of Sport Scientists in Professional Sport.” European Journal of Sport Science 21, no. 11: 1579–1587. 10.1080/17461391.2020.1842511. 33096969

[ejsc70007-bib-0003] Brislane, Á. , M. J. Hayman , and M. H. Davenport . 2022. “A Delphi Study to Identify Research Priorities Regarding Physical Activity, Sedentary Behavior and Sleep in Pregnancy.” International Journal of Environmental Research and Public Health 19, no. 5: 2909. 10.3390/ijerph19052909. 35270601 PMC8909963

[ejsc70007-bib-0004] Brocherie, F. , and A. Beard . 2020. “All Alone We Go Faster, Together We Go Further: The Necessary Evolution of Professional and Elite Sporting Environment to Bridge the Gap Between Research and Practice.” Frontiers in Sports and Active Living 2: 631147. 10.3389/fspor.2020.631147. 33585813 PMC7874745

[ejsc70007-bib-0005] Brooks, J. H. M. , C. W. Fuller , S. P. T. Kemp , and D. B. Reddin . 2005a. “Epidemiology of Injuries in English Professional Rugby Union: Part 1 Match Injuries.” British Journal of Sports Medicine 39, no. 10: 757–766. 10.1136/bjsm.2005.018135. 16183774 PMC1725032

[ejsc70007-bib-0006] Brooks, J. H. M. , C. W. Fuller , S. P. T. Kemp , and D. B. Reddin . 2005b. “Epidemiology of Injuries in English Professional Rugby Union: Part 2 Training Injuries.” British Journal of Sports Medicine 39, no. 10: 767–775. 10.1136/bjsm.2005.018408. 16183775 PMC1725038

[ejsc70007-bib-0007] Cardinale, M. 2017. “Commentary on ‘Towards a Grand Unified Theory of Sports Performance’.” Human Movement Science 56: 160–162. 10.1016/j.humov.2017.04.015. 28483216

[ejsc70007-bib-0008] Coutts, A. J. 2017. “Challenges in Developing Evidence‐Based Practice in High‐Performance Sport.” International Journal of Sports Physiology and Performance 12, no. 6: 717–718. 10.1123/IJSPP.2017-0455. 28832264

[ejsc70007-bib-0009] Cross, M. J. , R. Tucker , M. Raftery , et al. 2019. “Tackling Concussion in Professional Rugby Union: A Case‐Control Study of Tackle‐Based Risk Factors and Recommendations for Primary Prevention.” British Journal of Sports Medicine 53, no. 16: 1021–1025. 10.1136/bjsports-2017-097912. 29021244

[ejsc70007-bib-0010] Cross, M. J. , S. Williams , G. Trewartha , S. P. T. Kemp , and K. A. Stokes . 2016. “The Influence of In‐Season Training Loads on Injury Risk in Professional Rugby Union.” International Journal of Sports Physiology and Performance 11, no. 3: 350–355. 10.1123/ijspp.2015-0187. 26309331

[ejsc70007-bib-0011] Daneshvar, D. H. , E. S. Nair , Z. H. Baucom , et al. 2023. “Leveraging Football Accelerometer Data to Quantify Associations Between Repetitive Head Impacts and Chronic Traumatic Encephalopathy in Males.” Nature Communications 14, no. 1: 3470. 10.1038/s41467-023-39183-0. PMC10281995 37340004

[ejsc70007-bib-0012] Elo, S. , and H. Kyngäs . 2008. “The Qualitative Content Analysis Process.” Journal of Advanced Nursing 62: 107–115. 10.1111/j.1365-2648.2007.04569.x. 18352969

[ejsc70007-bib-0013] Finch, C. F. , S. Talpey , A. Bradshaw , T. Soligard , and L. Engebretsen . 2016. “Research Priorities of International Sporting Federations and the IOC Research Centres.” BMJ Open Sport & Exercise Medicine 2, no. 1: e000168. 10.1136/bmjsem-2016-000168. PMC5125424 27900197

[ejsc70007-bib-0014] Frisch, N. , P. Atherton , M. M. Doyle‐Waters , et al. 2020. “Patient‐Oriented Research Competencies in Health (PORCH) for Researchers, Patients, Healthcare Providers, and Decision‐Makers: Results of a Scoping Review.” Research Involvement and Engagement 6, no. 1: 4. 10.1186/s40900-020-0180-0. 32055415 PMC7011284

[ejsc70007-bib-0015] Fullagar, H. H. K. , L. D. Harper , A. Govus , R. McCunn , J. Eisenmann , and A. McCall . 2019. “Practitioner Perceptions of Evidence‐Based Practice in Elite Sport in the United States of America.” Journal of Strength & Conditioning Research 33, no. 11: 2897–2904. 10.1519/JSC.0000000000003348. 31453942

[ejsc70007-bib-0016] Fullagar, H. H. K. , A. McCall , F. M. Impellizzeri , T. Favero , and A. J. Coutts . 2019. “The Translation of Sport Science Research to the Field: A Current Opinion and Overview on the Perceptions of Practitioners, Researchers and Coaches.” Sports Medicine 49, no. 12: 1817–1824. 10.1007/s40279-019-01139-0. 31214978

[ejsc70007-bib-0017] Giacobbi, P. R. , A. Poczwardowski , and P. Hager . 2005. “A Pragmatic Research Philosophy for Sport and Exercise Psychology.” Sport Psychologist 19, no. 1: 18–31: Published Online First. 10.1123/tsp.19.1.18.

[ejsc70007-bib-0018] Giles, S. , D. Fletcher , R. Arnold , A. Ashfield , and J. Harrison . 2020. “Measuring Well‐Being in Sport Performers: Where Are We Now and How Do We Progress?” Sports Medicine 50, no. 7: 1255–1270. 10.1007/s40279-020-01274-z. 32103451 PMC7305091

[ejsc70007-bib-0019] Graneheim, U. H. , and B. Lundman . 2004. “Qualitative Content Analysis in Nursing Research: Concepts, Procedures and Measures to Achieve Trustworthiness.” Nurse Education Today 24, no. 2: 105–112. 10.1016/j.nedt.2003.10.001. 14769454

[ejsc70007-bib-0020] Health Promotion Glossary of Terms 2021. (accessed 4 September 2024). https://www.who.int/publications/i/item/9789240038349.

[ejsc70007-bib-0021] Hendricks, S. 2021. “Rethinking Innovation and the Role of Stakeholder Engagement in Sport and Exercise Medicine.” BMJ Open Sport & Exercise Medicine 7, no. 2: e001009. 10.1136/bmjsem-2020-001009. PMC8160180 34123408

[ejsc70007-bib-0022] Hendricks, S. , C. Emery , B. Jones , et al. 2023. “‘Tackling’ Rugby Safety Through a Collective Approach.” British Journal of Sports Medicine 57, no. 10: 562–563. 10.1136/bjsports-2023-107020. 37045555

[ejsc70007-bib-0023] Heyward, O. , S. Emmonds , G. Roe , S. Scantlebury , K. Stokes , and B. Jones . 2022. “Applied Sports Science and Sports Medicine in Women’s Rugby: Systematic Scoping Review and Delphi Study to Establish Future Research Priorities.” BMJ Open Sport & Exercise Medicine 8, no. 3: e001287. 10.1136/bmjsem-2021-001287. PMC9310180 35979431

[ejsc70007-bib-0024] Houser, J. , and K. S. Oman . 2010. Evidence‐Based Practice: An Implementation Guide for Healthcare Organizations. Jones & Bartlett Publishers.

[ejsc70007-bib-0025] Jones, B. , K. Till , S. Emmonds , et al. 2019. “Accessing Off‐Field Brains in Sport; an Applied Research Model to Develop Practice.” British Journal of Sports Medicine 53, no. 13: 791–793. 10.1136/bjsports-2016-097082. 28818959

[ejsc70007-bib-0026] Joshi, A. , S. Kale , S. Chandel , and D. Pal . 2015. “Likert Scale: Explored and Explained.” Journal Applied Science Technology 7, no. 4: 396–403. 10.9734/BJAST/2015/14975.

[ejsc70007-bib-0027] Lee, M. , M. Lancaster , L. Tulloch , et al. 2023. “Normative Isometric Plantarflexion Strength Values for Professional Level, Male Rugby Union Athletes.” Phys Ther Sport Off J Assoc Chart Physiother Sports Med 61: 114–121. 10.1016/j.ptsp.2023.03.007. 37003219

[ejsc70007-bib-0028] Morgan, D. L. 2007. “Paradigms Lost and Pragmatism Regained: Methodological Implications of Combining Qualitative and Quantitative Methods.” Journal of Mixed Methods Research 1: 48–76. 10.1177/2345678906292462.

[ejsc70007-bib-0029] Morgan, D. L. 2014. Integrating Qualitative and Quantitative Methods: A Pragmatic Approach. SAGE Publications Inc.

[ejsc70007-bib-0030] Pelletier, C. , C. Ross , K. Bailey , T. M. Fyfe , K. Cornish , and E. Koopmans . 2022. “Health Research Priorities for Wildland Firefighters: A Modified Delphi Study With Stakeholder Interviews.” BMJ Open 12, no. 2: e051227. 10.1136/bmjopen-2021-051227. PMC8814744 35115350

[ejsc70007-bib-0031] Roe, G. , S. Whitehead , L. Starling , et al. 2024. “Embracing the Impact From Instrumented Mouthguards (iMGs): A Survey of iMG Managers’ Perceptions of Staff and Player Interest Into the Technology, Data and Barriers to Use.” European Journal of Sport Science 24, no. 6: 670–681. 10.1002/ejsc.12101. 38874970 PMC11235837

[ejsc70007-bib-0032] Sawczuk T. , Cross M. , Owen C. , et al. The Application of Match‐Event and Instrumented Mouthguard (iMG) Data to Inform Match Limits: An Example Using Rugby Union Premiership and Rugby League Super League Data From England 2024. 10.1002/ejsc.12188 PMC11534626 39305464

[ejsc70007-bib-0033] Schwarz, E. , L. D. Harper , R. Duffield , et al. 2021. “Practitioner, Coach, and Athlete Perceptions of Evidence‐Based Practice in Professional Sport in Australia.” International Journal of Sports Physiology and Performance 16, no. 12: 1728–1735. 10.1123/ijspp.2020-0835. 34000715

[ejsc70007-bib-0034] Spranger, J. , A. Homberg , M. Sonnberger , and M. Niederberger . 2022. “Reporting Guidelines for Delphi Techniques in Health Sciences: A Methodological Review.” Z Evidenz Fortbild Qual Im Gesundheitswesen 172: 1–11. 10.1016/j.zefq.2022.04.025. 35718726

[ejsc70007-bib-0035] Stewart, W. , M. E. Buckland , B. Abdolmohammadi , et al. 2023. “Risk of Chronic Traumatic Encephalopathy in Rugby Union Is Associated With Length of Playing Career.” Acta Neuropathologica 146, no. 6: 829–832. 10.1007/s00401-023-02644-3. 37872234 PMC10627955

[ejsc70007-bib-0036] Stokes, K. A. , D. Locke , S. Roberts , et al. 2021. “Does Reducing the Height of the Tackle Through Law Change in Elite Men’s Rugby Union (The Championship, England) Reduce the Incidence of Concussion? A Controlled Study in 126 Games.” British Journal of Sports Medicine 55, no. 4: 220–225. 10.1136/bjsports-2019-101557. 31857335

[ejsc70007-bib-0037] van der Horst, N. , F. J. G. Backx , E. A. Goedhart , and B. M. A. Huisstede . 2017. “Return to Play After Hamstring Injuries in Football (Soccer): A Worldwide Delphi Procedure Regarding Definition, Medical Criteria and Decision‐Making.” British Journal of Sports Medicine 51, no. 22: 1583–1591. 10.1136/bjsports-2016-097206. 28360143

[ejsc70007-bib-0038] Verhagen, A. P. , V. H. C. de , R. A. de Bie , et al. 1998. “The Delphi List: A Criteria List for Quality Assessment of Randomized Clinical Trials for Conducting Systematic Reviews Developed by Delphi Consensus.” Journal of Clinical Epidemiology 51: 1235–1241. 10.1016/s0895-4356(98)00131-0. 10086815

[ejsc70007-bib-0039] West, S. W. , M. Cross , G. Trewartha , et al. 2021. “Trends in Match Concussion Incidence and Return‐To‐Play Time in Male Professional Rugby Union: A 16‐Season Prospective Cohort Study.” Brain Injury 35, no. 10: 1235–1244. 10.1080/02699052.2021.1972142. 34495819

[ejsc70007-bib-0040] West, S. W. , L. Starling , S. Kemp , et al. 2021. “Trends in Match Injury Risk in Professional Male Rugby Union: A 16‐Season Review of 10 851 Match Injuries in the English Premiership (2002‐2019): The Professional Rugby Injury Surveillance Project.” British Journal of Sports Medicine 55, no. 12: 676–682. 10.1136/bjsports-2020-102529. 33046453

[ejsc70007-bib-0041] West, S. W. , S. Williams , S. P. T. Kemp , et al. 2020. “Patterns of Training Volume and Injury Risk in Elite Rugby Union: An Analysis of 1.5 Million Hours of Training Exposure Over Eleven Seasons.” Journal of Sports Science 38, no. 3: 238–247. 10.1080/02640414.2019.1692415. 31755824

[ejsc70007-bib-0042] Williams, S. , E. Kay , R. Bryan , et al. 2023. “The Influence of Match Exposure on Injury Risk in Elite Men’s Rugby Union.” Journal of Science and Medicine in Sport 26, no. 1: 25–30. 10.1016/j.jsams.2022.10.016. 36371396

[ejsc70007-bib-0043] Williams, S. , G. Trewartha , S. P. T. Kemp , et al. 2017. “How Much Rugby Is Too Much? A Seven‐Season Prospective Cohort Study of Match Exposure and Injury Risk in Professional Rugby Union Players.” Sports Medicine 47, no. 11: 2395–2402. 10.1007/s40279-017-0721-3. 28361327 PMC5633632

[ejsc70007-bib-0044] Williams, S. , G. Trewartha , S. P. T. Kemp , et al. 2016. “Time Loss Injuries Compromise Team Success in Elite Rugby Union: A 7‐Year Prospective Study.” British Journal of Sports Medicine 50, no. 11: 651–656. 10.1136/bjsports-2015-094798. 26552415

[ejsc70007-bib-0045] Zambaldi, M. , I. Beasley , and A. Rushton . 2017. “Return to Play Criteria After Hamstring Muscle Injury in Professional Football: A Delphi Consensus Study.” British Journal of Sports Medicine 51, no. 16: 1221–1226. 10.1136/bjsports-2016-097131. 28246078

[ejsc70007-bib-0046] Zimmerman, K. A. , J. A. Hain , N. S. N. Graham , et al. 2024. “Prospective Cohort Study of Long‐Term Neurological Outcomes in Retired Elite Athletes: The Advanced BiomaRker, Advanced Imaging and Neurocognitive (BRAIN) Health Study Protocol.” BMJ Open 14, no. 4: e082902. 10.1136/bmjopen-2023-082902. PMC11043776 38663922

