# Supplementary material for: ‘We're All (Cauliflower) Ears’: A Delphi Study Including Staff and Players to Co‐Construct Sports Science and Medicine (Performance and Wellbeing) Research Priorities for Premiership Rugby
Source: Eur J Sport Sci. 2025 Jul 2;25(7):e70007. doi: 10.1002/ejsc.70007 (PMC12217044; doi:10.1002/ejsc.70007)
Supplement: Supplementary file 1 — Table S1 [file EJSC-25-e70007-s002.docx]

**Supplementary Table 1; Online questionnaire to establish research priorities of stakeholders**

The aim of this project is to establish the most important research priorities for clubs in the Premiership from the perspectives of staff and players. Based on your experiences playing in the Premiership, we would value your suggestions on research priorities in the areas of player wellbeing and performance (both defined below). This questionnaire will only take 10 minutes of your time to complete, and your contributions are greatly appreciated and will help to inform the future research strategy for Premiership Rugby.

**Staff**

Please provide your email address so that we can include you in future rounds.

What is your current job title?

What is your ethnicity?

What is your age (in years)?

What is your highest level of education achieved (e.g. GCSEs, A-levels, degree, master's degree, PhD)?

How many seasons have you worked in the **Premiership** at an **academy** level? (Staff)

How many seasons have you worked in **another professional league** at an **academy** level?

How many seasons have you worked in **international rugby union**at an **academy** level?

How many seasons have you worked in the **Premiership** at a **first team** level?

How many seasons have you worked in **another professional league** at a **first team**level?

How many seasons have you worked in **international rugby union** at a **first team** level?

**Players**

Please provide your email address so that we can include you in future rounds.

What is your playing position?

What is your ethnicity?

What is your age (in years)?

What is your highest level of education achieved (e.g. GCSEs, A-levels, degree, master's degree, PhD)?

How many seasons have you played in the**Premiership** at an **academy** level? (Player)

How many seasons have you played in **another professional league** at an **academy** level?

How many times have you been selected for an **international rugby union**match-day squad at **academy** level?

How many seasons have you played in the**Premiership** at a **first team** level?

How many seasons have you played in **another professional league** at a **first team**level?

How many times have you been selected for an **international rugby union** match-day squad at a **first team** level?

### **In the following section, you will be asked to provide up to 10 research priorities in the areas of wellbeing and performance. Please provide as much detail as possible for each suggested research priority.**

**WELLBEING RESEARCH PRIORITIES

Please list up to 10 specific player wellbeing research priorities that you feel are important for the Premiership.**

**Research definition:** The process of studying something to discover new information or reach a new understanding.  

**Wellbeing definition:** A positive state experienced by individuals. Similar to health, it is a resource for daily life and is determined by social, economic and environmental conditions. It encompasses quality of life, as well as the ability of people to contribute to the world in accordance with a sense of meaning and purpose (Adapted from WHO, (2021)). Components of wellbeing include Emotional (positive / negative emotional states e.g. happiness, stress, anxiety), Mental (e.g. purpose, resilience, achievement), Social (e.g. relationships, social integration / acceptance) Physical (e.g. general physical health, injuries, financial and living circumstances) (Adapted from Giles, (2020)).

**For example:** an investigation into the stressors experienced by players in the Premiership.

**PERFORMANCE RESEARCH PRIORITIES

Please list up to 10 specific player performance research priorities that you feel are important for the Premiership.**

**Research definition:** The process of studying something to discover new information or reach a new understanding.  

**Performance definition:** Anything that contributes to how well a player or team can compete in match-play.

**For example:** an investigation into the transfer of tackle technique training to match scenarios.
