# Supplementary material for: ‘We're All (Cauliflower) Ears’: A Delphi Study Including Staff and Players to Co‐Construct Sports Science and Medicine (Performance and Wellbeing) Research Priorities for Premiership Rugby
Source: Eur J Sport Sci. 2025 Jul 2;25(7):e70007. doi: 10.1002/ejsc.70007 (PMC12217044; doi:10.1002/ejsc.70007)
Supplement: Supplementary file 2 — Table S2 [file EJSC-25-e70007-s001.docx]

**Supplementary Table 2:** Research priorities that did not reach consensus and accompanied distribution (%) of votes across the three categories along with the median response and associated interquartile range (IQR).

| **Category** | **Higher-order research priority** | **Low-Medium-High**  **(%)** | **Median response (IQR)** |
| --- | --- | --- | --- |
| **THEME; WELLBEING** | | | |
| Staff capabilities | The competencies required of staff to appropriately manage the complexities of players' experiences (e.g., youth players, psychology of non-selection). | 5-27-68 | High  (M-H) |
| External stressors | The effects of external stressors (e.g., media, family, & friends) on player wellbeing | 11-24-65 | High  (M-H) |
| Fatigue & recovery | The effects of changing sleep routines (e.g., early/late training, sleeping in hotels) on sleep quality & wellbeing. | 11-43-46 | Medium  (M-H) |
| Medical | Player perceptions of, & contributing factors to, pain. | 16-41-43 | Medium  (M-H) |
| Medical | The use of prescription medication & legal psychoactive substances (e.g. snus, alcohol). | 11-46-43 | Medium  (M-H) |
| Training programme | Aspects of the training programme that may affect player wellbeing (e.g. training time of day, training day length, in-season breaks). | 11-46-43 | Medium  (M-H) |
| Life skills | Approaches to maximise development of life skills in players (e.g. financial knowledge, time management, skills & qualifications beyond rugby, activities of daily living). | 16-49-35 | Medium  (M-H) |
| Psychology | Personality profiling to improve understanding of self & others. | 22-43-35 | Medium  (M-H) |
| Psychology | Factors that limit or facilitate help-seeking behaviours. | 22-43-35 | Medium  (M-H) |
| Fatigue & recovery | Knowledge, perceptions & behaviours relating to recovery strategies | 14-59-27 | Medium  (M-H) |
| Selection | The effect of non-selection (e.g. consistently in non-23 or on loan) on wellbeing. | 19-54-27 | Medium  (M-H) |
| Socio-economic | The impact of financial & contract status on wellbeing & performance | 27-46-27 | Medium  (L-H) |
| External stressors | Positive & negative aspects of being a dual-career athlete, & the potential support mechanisms required. | 19-57-24 | Medium  (M-M) |
| Culture | Cultural factors that affect wellbeing (e.g. misogynistic, macho, banter vs bullying). | 30-46-24 | Medium  (L-M) |
| **THEME; PERFORMANCE** | | | |
| Transference | Transfer of physical training practices (e.g. speed, strength, COD) to position-specific match performance indicators. | 3-30-68 | High  (M-H) |
| Transference | The relationship between training type (e.g. units, skills) & associated characteristics (e.g. content, intensity, duration, time), with related match performance indicators. | 3-30-68 | High  (M-H) |
| Match characteristics | The association between match characteristics & winning performance. | 8-24-68 | High  (M-H) |
| Lifestyle factors | Lifestyle factors that may influence player performance (e.g. education, work-life balance, use of snus & alcohol, behaviours away from the training ground). | 3-32-65 | High  (M-H) |
| Transference | Different methods to maximise learning & transfer to match performance (e.g. drill vs. game-based training, coaching techniques, practice design, use of meetings). | 11-27-62 | High  (M-H) |
| Transference | The effects of playing in different competitions on the ability to play and perform in the Premiership (e.g. 7's, Championship, Nat 1). | 8-41-51 | High  (M-H) |
| Fatigue & recovery | The effectiveness of recovery strategies including, but not limited to; breathwork, cold water immersion, red light therapy, sleep. | 8-41-51 | High  (M-H) |
| Transference | The transfer of technical training practices (e.g. kicking, tackling) to associated match performance indicators. | 19-43-38 | Medium  (M-H) |
| Psychology | Players' perceptions of psychological support in the Premiership. | 16-46-38 | Medium  (M-H) |
| Training characteristics | Training characteristics (content, volume, intensity, time) across levels. Including, but not limited to; kicking, units, team, contact training, N-23 sessions, daily & weekly totals. | 32-32-35 | Medium  (L-H) |
| Transference | Use of pre-match / half-time strategies to maximise match performance. (e.g. specific warm-ups, primers). | 27-41-32 | Medium  (L-H) |
| Nutrition | Optimal pre-match nutritional strategies, including ergogenic aids. | 16-51-32 | Medium  (M-H) |
| Player availability | The association between player availability & performance. | 14-57-30 | Medium  (M-H) |
| Match characteristics | The differences in match characteristics between competitions (e.g. Sub-elite, university). | 24-51-24 | Medium  (M-M) |
| Fatigue & recovery | The benefits (e.g. social) & drawbacks (effect on recovery) of alcohol. | 41-38-22 | Medium  (L-M) |
| **THEME; INJURY** | | | |
| Risk factors | The association between training stimuli (e.g. prescription of sprints, completion of preseason training, contact training) & injury. | 8-41-51 | High  (M-H) |
| Risk factors | The association between biomechanics (e.g. running form, chronic ankle instability) & injury. | 14-46-41 | Medium  (M-H) |
| Risk factors | The impact of external stressors (e.g., family, study, contract status) on injury risk. | 30-38-32 | Medium  (L-M) |
